# Semantic Evaluation of Nursing Assessment Scales Translations by ChatGPT 4.0: A Lexicometric Analysis

**DOI:** 10.3390/nursrep15060211

**Published:** 2025-06-11

**Authors:** Mauro Parozzi, Mattia Bozzetti, Alessio Lo Cascio, Daniele Napolitano, Roberta Pendoni, Ilaria Marcomini, Elena Sblendorio, Giovanni Cangelosi, Stefano Mancin, Antonio Bonacaro

**Affiliations:** 1 Medicine and Surgery Department, University of Parma, Via Gramsci 14, 43126 Parma, Italy; mauro.parozzi@unipr.it (M.P.); antonio.bonacaro@unipr.it (A.B.); 2 Direction of Health Professions, ASST Cremona, 26100 Cremona, Italy; mattia.bozzetti@asst-cremona.it (M.B.); roberta.pendoni@asst-cremona.it (R.P.); 3 La Maddalena Cancer Center, 90146 Palermo, Italy; locascio.alessio@lamaddalenanet.it; 4 CEMAD, Fondazione Policlinico Gemelli, 00168 Rome, Italy; daniele.napolitano@policlinicogemelli.it; 5 Center for Nursing Research and Innovation, Vita-Salute San Raffaele University, 20132 Milan, Italy; marcomini.ilaria@unisr.it; 6 Azienda Ospedaliero—Universitaria Consorziale Policlinico di Bari, Piazza Giulio Cesare 11, 70124 Bari, Italy; sblendorioelena@gmail.com; 7 Experimental Medicine and Public Health Unit, School of Pharmacy, University of Camerino, 62032 Camerino, Italy; 8 IRCCS Humanitas Research Hospital, Via Manzoni 56, 20089 Rozzano, Italy

**Keywords:** semantics, nursing assessment, psychometrics, ChatGPT, artificial intelligence, lexicometric analysis

## Abstract

**Background/Objectives**: The use of standardized assessment tools within the nursing care process is a globally established practice, widely recognized as a foundation for evidence-based evaluation. Accurate translation is essential to ensure their correct and consistent clinical use. While effective, traditional procedures are time-consuming and resource-intensive, leading to increasing interest in whether artificial intelligence can assist or streamline this process for nursing researchers. Therefore, this study aimed to assess the translation’s quality of nursing assessment scales performed by ChatGPT 4.0. **Methods**: A total of 31 nursing rating scales with 772 items were translated from English to Italian using two different prompts, and then underwent a deep lexicometric analysis. To assess the semantic accuracy of the translations the Sentence-BERT, Jaccard similarity, TF-IDF cosine similarity, and Overlap ratio were used. Sensitivity, specificity, AUC, and AUROC were calculated to assess the quality of the translation classification. Paired-sample *t*-tests were conducted to compare the similarity scores. **Results**: The Maastricht prompt produced translations that are marginally but consistently more semantically and lexically faithful to the original. While all differences were found to be statistically significant, the corresponding effect sizes indicate that the advantage of the Maastricht prompt is slight but consistent across all measures. The sensitivity of the prompts was 0.929 (92.9%) for York and 0.932 (93.2%) for Maastricht. Specificity and precision remained for both at 1.000. **Conclusions**: Findings highlight the potential of prompt engineering as a low-cost, effective method to enhance translation outcomes. Nonetheless, as translation represents only a preliminary step in the full validation process, further studies should investigate the integration of AI-assisted translation within the broader framework of instrument adaptation and validation.

## 1. Introduction

The use of standardized assessment tools within the nursing care process is today a globally established practice and is widely recognized as a foundation for evidence-based evaluation [1]. The routine application of these tools enables nursing professionals to objectively assess patient conditions, significantly contributing to patient safety [2] and facilitating interprofessional communication [3].

These tools are often developed in English-speaking countries, and when adopted in other linguistic or cultural contexts, they must be translated and adapted to ensure semantic accuracy, clinical relevance, and psychometric validity in the local setting. Regardless of the context—whether national or international—the effective implementation of such tools depends not only on their availability in a language spoken by healthcare providers—who may not always be fully proficient in international English—but also on the need for legal clarity and comprehensibility by all stakeholders involved in the care process. This underscores the importance of using accurately translated and properly validated instruments [4]. Therefore, accurate translation is essential to ensure their correct and consistent clinical use. Traditionally, the translation and cultural adaptation of psychometric tools have involved rigorous methodologies, including forward-translation, back-translation, expert committee reviews, and cognitive debriefings [5]. While effective, these procedures are time-consuming and resource-intensive. For example, unless the researcher is also a professional translator, one or more translators must be involved, which increases costs and extends timelines. This has led to growing interest in whether artificial intelligence can assist or streamline this step as part of the broader validation process for nursing researchers. In light of these challenges and given the increasing demand for faster and more accessible solutions, attention has recently turned to the potential of emerging technologies to support the translation and adaptation of nursing assessment tools.

The use of machine translation tools is not new in the nursing field [6,7,8]; however, in recent years, advances in natural language processing (NLP) and the development of large language models (LLMs) have significantly expanded the potential for automated translation across diverse fields, including academia, healthcare, and cross-cultural research [9,10,11,12]. Among these models, ChatGPT 4.0 represents a state-of-the-art system capable of producing fluent and contextually appropriate translations, making it a promising tool for the adaptation of psychometric instruments across languages.

The process of translating psychometric instruments, however, is inherently complex. It requires more than literal translation; it demands careful preservation of semantic meaning, syntactic appropriateness, and cultural relevance to ensure that the instrument maintains its validity and reliability in the target context [5]. Even subtle shifts in meaning during translation can affect the factorial structure, internal consistency, or measurement invariance of a scale, ultimately compromising the comparability of results across languages.

Despite the impressive generative capabilities of large language models, there remains limited empirical evidence on how specific aspects of the translation process—such as semantic fidelity, grammatical correctness, and naturalness of expression—are influenced by the design of user prompts. Recent research on prompt engineering suggests that the specificity and structure of prompts can meaningfully affect model outputs, including in tasks requiring high linguistic precision [11,12].

The central hypothesis of this study is that artificial intelligence—specifically, ChatGPT 4.0—can support and potentially streamline the translation of nursing assessment tools while maintaining high levels of semantic and lexical fidelity. By evaluating the quality of translations generated through different prompt designs, this study seeks to determine whether prompt engineering can improve the reliability of AI-assisted translation in the context of nursing research.

## 2. Materials and Methods

The primary objective of this study was to evaluate the translation quality of nursing assessment scales performed by ChatGPT 4.0, using two publicly available prompts developed by York and Maastricht Universities (Table 1). To the best of the authors’ knowledge, there are no readily available checklists for reporting, so the Clinical Artificial Intelligence Research Checklist Proposal [13] was followed for reporting this study.

The choice of the two prompting strategies in this study was based on their alignment with widely accepted translation practices in cross-cultural research. The York prompt seems designed to reflect a standard, general-purpose translation instruction, emphasizing the preservation of meaning and context but without explicitly guiding grammatical or stylistic aspects. In contrast, the Maastricht prompt incorporated a more detailed set of instructions, focusing not only on semantic fidelity but also on grammatical correctness, fluency, handling of proper nouns, and maintenance of the original tone and style. This distinction was intended to simulate two common real-world approaches: minimal guidance versus comprehensive guidance, both of which are frequently employed in translation workflows [14,15].

While other prompting strategies could theoretically have been adopted—such as instructing the model to produce a literal translation, a culturally adapted version, or a translation optimized for readability—the York and Maastricht prompts were selected because they represent two practical and widely applicable ends of the translation instruction spectrum. Minimal prompts are often preferred for efficiency, whereas detailed prompts are increasingly advocated in fields where linguistic and conceptual precision are critical [14,16].

Moreover, recent literature on prompt engineering suggests that overly complex or excessively prescriptive prompts may not necessarily yield better outputs and could introduce variability depending on how the model interprets intricate instructions [11].

### 2.1. Database Building

In April 2025, the databases CINAHL, PubMed, and Scopus were searched for nursing assessment scales validated in Italian. The search terms used were “Nurs*”, “Scale”, “Tool”, and “Italian validation”, combined using the Boolean operators AND and OR. Additionally, a convenience sample of five Italian nursing researchers was consulted to identify any translated and validated tools or relevant articles that may not have been captured in the initial database search. No date restrictions were applied.

Eligibility Criteria included the following: Articles published in either Italian or English. The original scales language had to be English, and the translated version had to be in Italian (authors’ native language); the article had to be available as full free text; and the Italian version of the scale had to be included within the published article or provided by the authors. 

Exclusion Criteria were as follows: articles involving cultural translations or adaptations that, in the judgment of the research team, resulted in major structural modifications—such as the addition or removal of items or reorganization of domains—that could compromise comparability with the original instrument.

The articles and scales were assessed independently by two researchers. In cases of discordance, the opinion of a third researcher was sought. To perform the appropriate tests, we first collected the original scales in the native language (English) alongside their translation in the target language (Italian); the published translation into the target language was considered the reference standard. The original scales were then processed using CHatGPT 4.0 with the aforementioned prompts, developed by the university of York [17] and Maastricht [18], to compare how each prompt influenced the quality of the translations, comparing them to the reference standard (the published translated scale). Prompts were used in their original language (English). Translations and analysis were performed in April 2025.

### 2.2. Lexicometric Analysis

In order to assess the semantic accuracy of the translations, various similarity metrics were used, starting with SBERT (Sentence-BERT, a transformer-based model that generates dense vector representations of sentences, capturing their semantic meaning) [19]. The advantage of SBERT over traditional methods lies in its ability to consider context when comparing sentence similarity, making it especially useful for evaluating machine-generated translations. By comparing the SBERT scores of the original sentence with those of the translated sentences, we were able to determine how closely the translations aligned with the original in terms of semantic meaning.

In addition to SBERT, other traditional metrics were also used for comparison. These included Jaccard similarity [20], TF-IDF cosine similarity [21], and Overlap ratio. Each of these metrics has its specific advantages. Jaccard similarity measures the proportion of shared words between two sets, providing a straightforward evaluation of how much overlap exists between the original and translated text. A cutoff value of 0.3 to 0.5 was used for Jaccard similarity. Lower values indicate less overlap and potentially lower translation quality, while higher values suggest a closer match. The choice of this range is supported by works such as Jaccard (1901) [20] where values between 0.3 and 0.5 typically indicate a moderate overlap in content, which is often found in machine translation tasks.

TF-IDF cosine similarity evaluates the semantic similarity between two texts based on their term frequency (TF) and inverse document frequency (IDF). It weighs the importance of words in relation to their frequency within a document and across a corpus. A cutoff range of 0.6 to 0.8 was selected for TF-IDF cosine similarity, as values in this range are typically considered indicative of a close semantic match between the original and translated sentences. Research by Salton and Buckley (1988) [22] and Manning et al. (2008) [23] indicates that TF-IDF values in this range effectively capture the relevance of terms while minimizing the impact of common, less informative words.

Overlap ratio quantifies the percentage of shared n-grams (sequences of n words) between the original and translated text. This metric emphasizes the degree to which the same sequences of words appear in both texts. A cutoff range of 0.6 to 0.8 was used for Overlap ratio, as higher values indicate greater similarity in n-gram sequences, which correlates with higher translation accuracy. The utility of n-gram overlap in machine translation evaluation has been well-documented in studies such as Papineni et al. (2001) [24], where higher n-gram overlap between the source and target texts generally correlates with better translation quality.

The SBERT model, specifically, was used due to its ability to generate dense vector representations that capture the semantic meaning of sentences. It has been shown in Reimers and Gurevych (2019) [19] that SBERT outperforms traditional methods in capturing the semantic similarity between sentences, making it especially useful for tasks like machine translation evaluation. A cutoff of 0.85 for SBERT was chosen based on the work of Devlin et al. (2019) [25], who demonstrated that cosine similarity scores of 0.85 or higher typically indicate a high-quality match in terms of semantic meaning, particularly in tasks where fine-grained semantic distinctions are critical.

The selection of these metrics and cutoff thresholds was informed not only by prior research in natural language processing, but also by the linguistic and clinical demands of nursing-specific instruments. In this context, preserving semantic fidelity is essential, as minor lexical shifts may lead to misinterpretation of clinical constructs or affect the psychometric properties of a scale. The chosen thresholds reflect conservative standards for translation accuracy, ensuring that the AI-generated versions maintain terminological consistency and conceptual clarity in line with the expectations of healthcare professionals and researchers.

These metrics, when used in conjunction, provide a comprehensive approach to evaluating the quality of translations, allowing for both fine-grained semantic comparisons (through SBERT) and traditional syntactic and lexical evaluations (through Jaccard, TF-IDF, and Overlap).

To classify translations as semantically correct or incorrect, an SBERT cutoff threshold of 0.85 was applied, which is commonly used in natural language processing to indicate a high level of semantic similarity. A translation with an SBERT cosine similarity score of 0.85 or higher was considered a correct translation, while scores below this threshold indicated potential issues with the semantic accuracy of the translation.

Finally, once the translations were classified based on the 0.85 cutoff, several performance metrics were calculated to assess the quality of the translation classifications. These metrics included sensitivity, specificity, AUC (Area Under the Curve), and AUROC (Area Under the Receiver Operating Characteristic Curve). Sensitivity measures the proportion of true positives (correct translations) identified by the model, while specificity evaluates the proportion of true negatives (incorrect translations) correctly identified. AUROC was used to evaluate the overall ability of the model to distinguish between correct and incorrect translations across various classification thresholds. Analyses were conducted using Python 3.13.

### 2.3. Semantic Subgroup Analysis

In this study, a semantic subgroup analysis was conducted to assess the quality of translations based on linguistic features that could significantly influence the results. The data were divided into distinct groups according to specific characteristics of the sentences in the dataset.

Based on some concerns expressed in the literature regarding the length of the sentences to be translated [15], sentences were categorized based on length into three groups: short, medium, and long. Sentence length was defined by the number of words, with short sentences containing fewer than 10 words, medium sentences ranging from 10 to 20 words, and long sentences containing more than 20 words. This classification was made to determine whether sentence length affects translation quality, as shorter sentences are generally easier to translate accurately compared to longer, more complex ones.

Sentences were also divided based on the presence of negation, with sentences containing negations (e.g., “not”, “no”, “never”) classified as having negation and those without as not having negation [26]. Negation is a critical linguistic feature that alters the meaning of a sentence, and this division allows for the examination of how negation influences translation accuracy [27].

Additionally, sentences were categorized based on the presence of intensifiers, such as “very”, “extremely”, or “incredibly”. Sentences containing these intensifiers were classified as having intensity, while others were classified as lacking intensity. Intensifiers can significantly affect the meaning of a sentence, and their presence or absence could influence the semantic accuracy of translations [28].

### 2.4. Advanced Embedding-Based Consistency Metrics

Recent reviews by Sblendorio et al. (2024) [29] and Galli et al. (2024) [30] have identified MPNet v2 as the state-of-the-art technique for measuring text similarity and evaluating Large Language Model response consistency over time.

While Dash et al.’s Stanford study [31] introduced cosine and Jaccard similarity for consistency assessment of Large Language Model responses to clinical questions, these traditional metrics have significant limitations. They found low similarity scores when testing large language models with identical questions over time (average Jaccard similarity: 0.27 for GPT-3.5, 0.29 for GPT-4; average cosine similarity: 0.36 for GPT-3.5, 0.45 for GPT-4).

However, Jaccard and cosine similarity primarily analyze surface-level text features, comparing shared words and word frequency vectors without capturing underlying meanings and context. For more accurate assessment, semantic analysis methods using transformer models like MPNet v2 are more appropriate.

MPNet v2 (all-mpnet-base-v2 on HuggingFace) is a transformer that compresses input text into a 768-dimensional vector containing both syntactic and semantic information. This approach preserves relationships and provides understanding beyond simple word patterns, capturing nuanced meanings that traditional metrics miss.

Based on comprehensive reviews, MPNet v2 represents the current best practice for comparing text similarity and evaluating Large Language Model response consistency over time. When testing Large Language Model consistency, researchers should close previous sessions before resubmitting questions to avoid context influence, rather than focusing on specific time intervals between submissions. For reproducible results, MPNet v2 is available at https://huggingface.co/sentence-transformers/all-mpnet-base-v2 (accessed on 18 April 2025).

## 3. Results

A total of N.31 instruments with a total of 772 items were analyzed. The following table (Table 2) summarizes statistics for each metric.

A series of paired-sample *t*-tests was conducted to compare the similarity scores produced by the York and Maastricht prompts relative to the human gold standard, across four distinct metrics: SBERT cosine similarity, Jaccard similarity, TF-IDF cosine similarity, and Overlap ratio. The results revealed that Maastricht consistently outperformed York across all metrics. Specifically, SBERT scores were statistically significant for Maastricht (t = −2.223, *p* = 0.0265), although the difference in means was relatively small (0.819 vs. 0.814). Jaccard similarity also favored Maastricht (t = −3.953, *p* = 0.0001), with a higher average score (0.430 compared to 0.413). Likewise, TF-IDF cosine similarity was statistically significant for Maastricht (t = −3.852, *p* = 0.0001), suggesting that its lexical output aligned more closely with the gold standard (0.495 vs. 0.478). Finally, the overlap ratio analysis confirmed the same pattern (t = −3.843, *p* = 0.0001), with Maastricht again exhibiting higher average similarity (0.574 vs. 0.559). These findings suggest that the Maastricht prompt yields translations that are marginally but consistently more semantically and lexically faithful to the original reference, compared to those generated by the York prompt.

**Figure 1:**
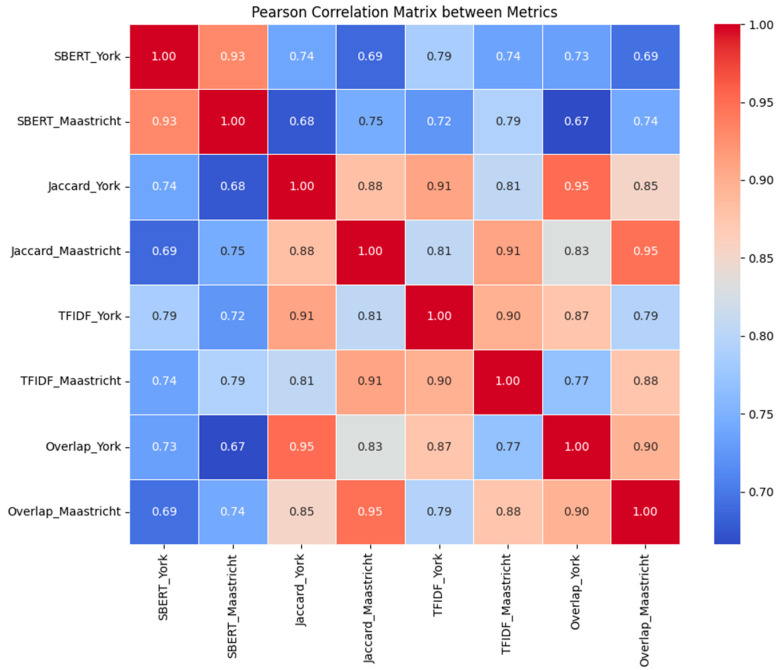
Correlation matrix between similarity indexes. All the values are statistically significant at p < 0.0001.

To assess the magnitude of the differences between the York and Maastricht prompts across similarity metrics, Cohen’s *d* was computed for each paired comparison. Results revealed consistently small effect sizes in favor of Maastricht: *d* = −0.080 for SBERT similarity, *d* = −0.142 for Jaccard similarity, *d* = −0.139 for TF-IDF cosine similarity, and *d* = −0.138 for word Overlap ratio. While all differences were statistically significant, the corresponding effect sizes indicate that the advantage of the Maastricht prompt is small in magnitude, yet consistent across all measures. The Pearson correlation matrix supported the use of SBERT as an effective metric for translation evaluation, as it aligns well with traditional lexical-based measures, as seen in Figure 1, correlation matrix.

### 3.1. Predictive Performance

**Figure 2:**
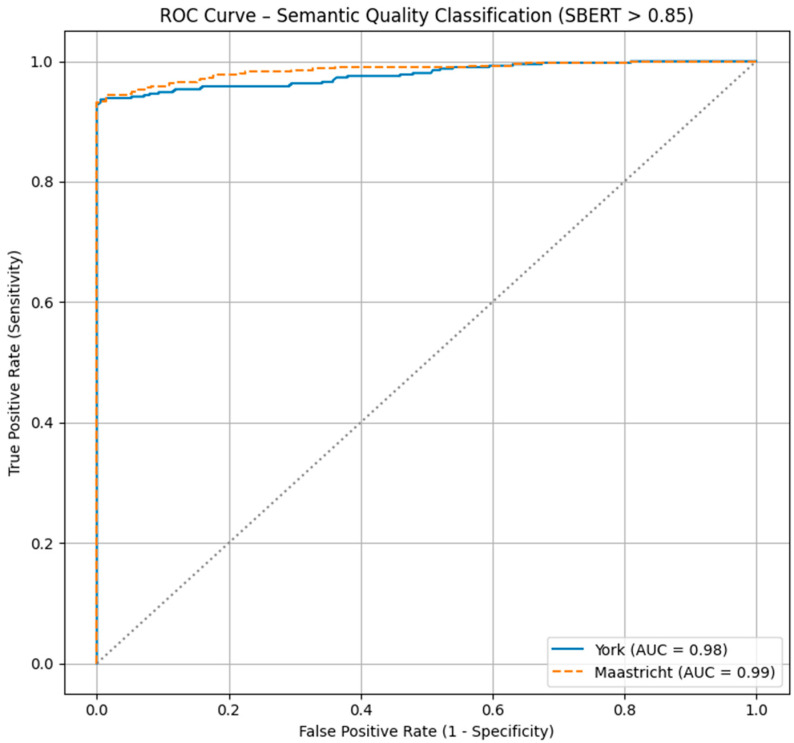
Discriminant performance of translation methods.

The York prompt yielded a sensitivity (recall) of 0.929, indicating that the model correctly identified 92.9% of semantically accurate translations. The specificity was 1.000, meaning that all incorrect translations were correctly identified. The precision was also 1.000, ensuring that every translation classified as correct was semantically accurate. The accuracy of the York prompt was 0.962, and the F1 score, which balances precision and recall, was 0.963. For the Maastricht prompt, the sensitivity slightly improved to 0.932, with the model correctly identifying 93.2% of semantically accurate translations. As with York, the specificity and precision remained perfect at 1.000, indicating no false positives. The accuracy for Maastricht was 0.964, with the F1 score slightly higher at 0.965 compared to York. These results are summarized in Table 3. Prompt predictive performance can be seen in Figure 2.

### 3.2. Subgroup Analysis

The semantic accuracy of translations was evaluated using SBERT scores with a threshold of 85% for identifying semantically correct translations. The analysis was conducted across several subgroups, considering variables such as sentence length, the presence of negations, and the presence of intensity markers. The following table presents the results for each subgroup in terms of SBERT scores (Table 4).

### 3.3. Sentence Length

For short sentences (n = 539), the SBERT score for York was 0.799, and for Maastricht it was 0.805. The percentage of translations above the 85% similarity threshold was 0.443 for York and 0.456 for Maastricht. Cohen’s d indicated a small effect size of −0.110, with a statistically significant difference between the two prompts, as evidenced by the *t*-test (t = −2.546, *p* = 0.011).

In the medium-length sentence group (n = 205), the SBERT score for York was 0.855, and for Maastricht it was 0.853. The percentage of translations above the 85% threshold was 0.639 for York and 0.600 for Maastricht. Cohen’s d indicated a negligible effect size of 0.060, with no statistically significant difference between the two prompts, as the *t*-test (t = 0.861, *p* = 0.390) resulted in a non-statistically significant *p*-value.

For long sentences (n = 28), the SBERT score for York was 0.820, and for Maastricht it was 0.827. The percentage of translations above 85% similarity was 0.357 for York and 0.429 for Maastricht. Cohen’s D indicated a small effect size of −0.160, with no statistically significant difference found between the two prompts (t = −0.844, *p* = 0.406).

### 3.4. Presence of Negations

For sentences with negation (n = 37), the SBERT score for both York and Maastricht was similar, with values of 0.853 and 0.856, respectively. The percentage of translations above the 85% threshold was 0.676 for York and 0.595 for Maastricht. Cohen’s d indicated a negligible effect size of −0.070, and no statistically significant difference was found (t = −0.425, *p* = 0.674).

For sentences without negation (n = 735), the SBERT score for York was 0.812, and for Maastricht it was 0.817. The percentage of translations above 85% similarity was 0.483 for York and 0.488 for Maastricht. Cohen’s d was −0.080, indicating a small effect size, with a statistically significant difference between the two prompts (t = −2.181, *p* = 0.030).

### 3.5. Presence of Intensity

For sentences without intensity markers (n = 772), the SBERT score for York was 0.814, and for Maastricht it was 0.819. The percentage of translations above 85% similarity was 0.492 for York and 0.494 for Maastricht. Cohen’s d indicated a small effect size of −0.080, with a statistically significant difference between the prompts (t = −2.223, *p* = 0.027).

## 4. Discussion

This study examined the quality of the translation of nursing assessment scales performed by ChatGPT 4.0, a usable and freely accessible artificial intelligence. Two distinct public-domain prompting strategies (York and Maastricht) were applied to the translation of psychometric instrument items from English to Italian.

Across all semantic and lexical similarity metrics evaluated, the Maastricht prompt consistently produced translations closer to the human translated published version (reference standard). Although the absolute differences between the prompts were statistically significant but small, the findings highlight that even slight modifications in prompt design can impact machine translation outcomes. To our knowledge, this is among the first studies systematically comparing the effect of prompt engineering on the translation quality of structured psychometric materials using advanced semantic evaluation techniques [11,32].

The results demonstrate that detailed and structured prompts, like Maastricht, can enhance the semantic fidelity of translations generated by large language models. The small but consistent advantage observed suggests that explicit instructions on grammar, fluency, and technical precision help the model produce outputs that better preserve the original meaning, tone, and style of the source text. These findings align with recent research emphasizing the critical role of prompt specificity in optimizing large language model performance across tasks [9,12].

From a semantic perspective, the use of a model such as SBERT for evaluation offers key insights into the nature of the improvements observed. The slightly higher semantic similarity scores associated with the Maastricht prompt imply a better preservation of conceptual relationships and nuanced meanings, which is critical when translating clinical or assessment materials. This is particularly relevant for the Italian language, where grammatical structures, such as gender agreement, complex verb conjugations, and the richness of subordinate clauses, create additional layers of complexity compared to English [33]. A detailed prompt likely encouraged ChatGPT to be more attentive to these linguistic nuances, ensuring that translations not only matched the meaning but also conformed to natural Italian syntax and stylistic conventions.

The nature of the translated material—instrument items designed for psychometric assessment—further underscores the importance of semantic precision. Unlike creative writing or casual communication, the translation of assessment scales and structured assessment tools requires an extremely high degree of semantic equivalence to preserve the validity and reliability of the instrument across languages [16]. Even minor deviations in meaning can alter the tool’s psychometric properties, such as its factorial structure or measurement invariance [14]. Therefore, while the absolute differences between York and Maastricht were minor, the consistent advantage of the latter supports the notion that prompt refinement is a low-cost but effective strategy to safeguard semantic fidelity in the translation of structured evaluation materials.

Additionally, subgroup analyses revealed that prompt advantages were more evident in shorter sentences. This may be due to the fact that shorter items offer less contextual information, increasing the risk of misinterpretation if the translation approach is not sufficiently rigorous [14,15,34]. The finding that sentence complexity, such as negations or intensifiers, did not markedly influence translation quality suggests that ChatGPT 4.0 is robust in managing such linguistic features, consistent with previous reports of its performance in fine-grained semantic tasks [32]. This study supports the cautious use of ChatGPT in the translation processes of validated nursing instruments, suggesting its use as a support to the work of human translators. In particular, in procedures that involve the comparison of multiple translated versions, a version generated by ChatGPT may be used alongside a human translation to enrich the process of linguistic comparison and evaluation.

## 5. Limitations

Despite the promising results of this study, several limitations should be acknowledged. First, while automated semantic metrics offer a reliable and scalable approach to evaluating translation quality, they may fail to capture critical aspects of cultural adaptation, such as idiomatic usage, contextual appropriateness, and shifts in register. These elements are essential for ensuring conceptual equivalence in cross-cultural research contexts and typically require human expertise to address effectively [34,35]. A second limitation relates to the scarcity of items containing linguistic intensifiers—such as adverbs or adjectives that modify the strength or emotional tone of a statement (e.g., “very”, “extremely”, “highly”). Although the study initially aimed to assess how such features influence translation fidelity, the limited presence of intensifiers in the corpus reduced the statistical power of subgroup analyses. Given that intensifiers can significantly affect the perceived meaning of psychometric items, particularly in instruments measuring subjective experiences or attitudes, future research should more directly investigate their role in AI-assisted translation.

Third, although English and Italian belong to different language families (Germanic and Romance, respectively), their shared lexical heritage—due in part to historical Latin influences on English—may have facilitated higher-than-expected semantic alignment. This linguistic proximity could have positively biased the translation results, potentially inflating the perceived effectiveness of the prompts. As such, caution is advised when generalizing these findings to language pairs with greater structural or lexical divergence.

Finally, this study did not conduct a subgroup analysis based on nursing specialties. Given that fields such as intensive care, pediatrics, and anesthesiology often employ highly specialized terminology, it is possible that the accuracy of AI-generated translations varies across subspecialties. Future studies should explore whether prompt performance remains consistent across domains with distinct technical language, in order to better understand the contextual robustness of automated translation systems in nursing research.

## 6. Conclusions

The present study demonstrates that prompt design can meaningfully influence the translation quality of psychometric instrument items produced by large language models such as ChatGPT 4.0. Detailed prompts, such as the Maastricht strategy, yielded marginal but consistent improvements in semantic and lexical fidelity compared to more general instructions. These findings highlight the potential of prompt engineering as a low-cost, effective method to enhance translation outcomes, particularly in structured domains where semantic precision is critical, like nursing assessment scales, and suggest that nursing researchers may cautiously consider involving ChatGPT 4.0 in the translation procedures of instruments from English to Italian. However, while automated semantic similarity metrics efficiently evaluate translation performance, they cannot fully capture the subtleties of meaning, cultural adaptation, and linguistic appropriateness required in sensitive contexts. Future studies should integrate human expert review alongside automated assessments, particularly when translations are intended for use in clinical or research settings with high stakes of semantic equivalence.

## Figures and Tables

**Table 1:** Freely available prompts from York and Maastricht University (modified in the original and target language field).

Name	Prompt
York (York University)	Translate the following document from English to Italian, ensuring that the translation maintains the original meaning and context. Pay special attention to technical terms and industry-specific jargon to ensure accuracy and consistency.
Maastricht (Maastricht University)	Above, you see a text in English language. Please translate it to Italian language. Do not print the original text, just the translation. Follow the following instructions: Ensure the translation accurately reflects the original text’s meaning. The translation should have correct grammar, including proper sentence structure, verb conjugation, punctuation, and the correct use of articles. The translation should read naturally and fluently as if originally written in the target language. Avoid awkward phrasing or literal translations that sound unnatural. Pay special attention to proper nouns and specific terms. Names of people, places, organizations, and other terms that should not be translated must be handled with care to maintain their original meaning and recognition. Ensure that the translation maintains the original text’s tone and style.

**Table 2:** Prompt comparison.

	York	Maastricht	*p*-Value
SBERT M(SD)	0.814 (0.137)	0.819 (0.137)	0.026
Jaccard M(SD)	0.413 (0.238)	0.430 (0.247)	>0.001
TF-IDF M(SD)	0.478 (0.262)	0.495 (0.269)	>0.001
Overlap M(SD)	0.559 (0.246)	0.574 (0.246)	>0.001

**Table 3:** Predictive performance.

Metric	York	Maastricht
Sensitivity	0.93	0.932
Specificity	1	1
Precision	1	1
Accuracy	0.96	0.964
F1 Score	0.96	0.965

**Table 4:** Subgroup SBERT scores.

	SBERT		SBERT > 85%				
Group	York	Maastricht	York	Maastricht	Cohen’s D	Student t	*p*-Value
**Length**							
Short (n = 539)	0.799	0.805	0.443	0.456	−0.110	−2.546	**0.011**
Medium (n = 205)	0.855	0.853	0.639	0.600	0.060	0.861	0.390
Long (n = 28)	0.820	0.827	0.357	0.429	−0.160	−0.844	0.406
**Negations**							
True (n = 37)	0.853	0.856	0.676	0.595	−0.070	−0.425	0.674
False (n = 735)	0.812	0.817	0.483	0.488	−0.080	−2.181	**0.030**
**Intensity (false) (n = 772)**	0.814	0.819	0.492	0.494	−0.080	−2.223	**0.027**

Note: bold values are statistically significant comparisons.

## Data Availability

Full data are available upon reasonable request to the corresponding author. Dataset is publicly available on Mendeley Data at DOI: 10.17632/dbvtbcm59k.1.

## Public Involvement Statement

No public involvement in any aspect of this research.

## Guidelines and Standards Statement

To the best of author’s knowledge, there are not readily available checklists for reporting bibliometric analysis on the Equator Network; for this reason, the Clinical Artificial Intelligence Research Checklist Proposal [13] was followed for reporting this study.
