# Correction: Parozzi et al. Semantic Evaluation of Nursing Assessment Scales Translations by ChatGPT 4.0: A Lexicometric Analysis. *Nurs. Rep.* 2025, *15*, 211

**DOI:** 10.3390/nursrep15070251

**Published:** 2025-07-10

**Authors:** Mauro Parozzi, Mattia Bozzetti, Alessio Lo Cascio, Daniele Napolitano, Roberta Pendoni, Ilaria Marcomini, Elena Sblendorio, Giovanni Cangelosi, Stefano Mancin, Antonio Bonacaro

**Affiliations:** 1 Medicine and Surgery Department, University of Parma, Via Gramsci 14, 43126 Parma, Italy; mauro.parozzi@unipr.it (M.P.);; 2 Direction of Health Professions, ASST Cremona, 26100 Cremona, Italy; 3 La Maddalena Cancer Center, 90146 Palermo, Italy; 4 CEMAD, Fondazione Policlinico Gemelli, 00168 Rome, Italy; 5 Center for Nursing Research and Innovation, Vita-Salute San Raffaele University, 20132 Milan, Italy; 6 Azienda Ospedaliero—Universitaria Consorziale Policlinico di Bari, Piazza Giulio Cesare 11, 70124 Bari, Italy; 7 Experimental Medicine and Public Health Unit, School of Pharmacy, University of Camerino, 62032 Camerino, Italy; 8 IRCCS Humanitas Research Hospital, Via Manzoni 56, 20089 Rozzano, Italy

Elena Sblendorio was not included as an author in the original publication [1]. The corrected Author Contributions statement appears here. The authors state that the scientific conclusions are unaffected. This correction was approved by the Academic Editor. The original publication has also been updated.

**Author Contributions:** Conceptualization, M.P. and G.C.; Methodology, M.B. and M.P.; Formal Analysis, M.B. and M.P.; Investigation, R.P. and S.M.; Resources, G.C., E.S. and S.M.; Data Curation, D.N. and A.L.C.; Writing—Original Draft Preparation, I.M. and A.B.; Writing—Review and Editing, E.S.; Supervision, A.B.; Project Administration, M.P. and A.B. All authors have read and agreed to the published version of the manuscript.
